# Office-based physical activity: mapping a social ecological model approach against COM-B

**DOI:** 10.1186/s12889-020-8280-1

**Published:** 2020-02-03

**Authors:** Yasmin F. van Kasteren, Lucy K. Lewis, Anthony Maeder

**Affiliations:** 1 Flinders Digital Health Research Centre, Flinders University, GPO Box 2100, Adelaide, South Australia 5001 Australia; 2 Caring Futures Institute, College of Nursing and Health Sciences, Flinders University, GPO Box 2100, Adelaide, South Australia 5001 Australia

**Keywords:** Physical activity, Sedentary behaviour, Office work, Social ecological model, COM-B

## Abstract

**Background:**

There are growing concerns over the health impacts of occupational sedentary behaviour on office-based workers and increasing workplace recognition of the need to increase physical activity at work. Social ecological models provide a holistic framework for increasing opportunities for physical activity at work. In this paper we propose a social ecological model of office-based physical activity and map it against the Capability Motivation Opportunity (COM-B) framework to highlight the mechanisms of behaviour change that can increase levels of physical activity of office-based workers.

**Discussion:**

The paper proposes a social ecological model of physical activity associated with office-based settings. The model considers opportunities for both incidental and discretionary activities, as well as macro and micro factors on both socio-cultural and physical dimensions. The COM-B framework for characterising behaviour change interventions is used to highlight the underlying mechanisms of behaviour change inherent in the model.

**Summary:**

The broad framework provided by social ecological models is important for understanding physical activity in office-based settings because of the non-discretionary nature of sedentary behaviour of office-based work. It is important for interventions not to rely on individual motivation for behaviour change alone but to incorporate changes to the broader social ecological and physical context to build capability and create opportunities for more sustainable change.

## Background

There is a large body of evidence supporting the importance of physical activity for health and wellbeing. Sedentary behaviour is defined as “any waking behaviour characterized by an energy expenditure ≤1.5 metabolic equivalents (METs), while in a sitting, reclining or lying posture” [1]. Sedentary behaviour has been linked to increased risk of cardiovascular disease, cancer, weight gain, obesity and musculoskeletal pain [2–5]. For office workers, the nature of their work increases exposure to sedentary behaviour inherent in desk bound and increasingly computer related work activities [6]. For office workers, as much as 77% of time at work is spent in sedentary behaviour, and this is often accumulated in prolonged bouts [7, 8]. Both employers and employees have a vested interest in building opportunities for physical activity into their workday and their work place activities. Workplace initiatives involving physical exercise, lifestyle and ergonomics can improve employee wellbeing, their ability to perform their work tasks and productivity through reduced sick leave [9].

Over the last 15 years there has been an increasing push by health authorities and workplace health and safety to reduce the health risks associated with sedentary behaviour at work by increasing physical activity of office workers and by breaking up prolonged bouts of sedentary behaviour [10–15].

Current guidelines recommend that adults should engage in at least 20 min a day (or 150 min a week) of moderate to vigorous physical activity (MVPA) preferably in bouts of 10 min or more [16–18]. Health guidelines also recommend reducing sedentary behaviour, in particular prolonged bouts of sedentary behaviour. In Australia, guidelines for safety at work recommend taking a break from sitting at least every 30 min [11]. Importantly, the health risks from prolonged sedentary behaviour are independent of the risks associated with recommended levels of moderate to vigorous physical activity [15, 19]. In this article we use two complementary frameworks for identifying the opportunities and constraints for increasing physical activity to help office workers meet physical activity guidelines: Social ecological models and the COM-B framework [20–22].

### Social ecological model

Social ecological models have been widely used in health promotion since the 1980’s but only more recently for understanding the correlates of physical activity [21, 23, 24]. The value of using social ecological models is the holistic approach which identifies the agency of social, cultural, and environmental factors on health behaviours [22]. Social ecological models have proven to be an effective framework for understanding and guiding population-based health behaviour change interventions [22, 25–27]. Social ecological models reveal the multi-dimensional, multi-level, often nested layers of context [21, 28], the complexities of which are well explained in Stokols [25]. Levels of agency in social ecological models range from the individual to global, and dimensions include both socio-cultural and physical or environmental factors [27, 29].

Social ecological models can also be scaled down to focus on specific settings and contexts [23, 30]. Health settings are defined as “the place or social context in which people engage in daily activities in which environmental, organisational and personal factors interact to affect health and wellbeing” [31] [32]. The *settings* approach is a social ecological model developed for informing public health promotion and policy [30, 32]. The specificity and predictive capacity of social ecological models can be improved if research based on social ecological models focuses on both specific behaviours and specific contexts [23, 33]. Context refers to the locale or environment in which physical activity occurs, because people behave differently in different circumstances [23, 33]. This more targeted context-based approach can provide a better understanding of the interrelationships between the diverse correlates of physical activity and differences in the performance of specific behaviours in context [33]. The advantages of targeted context-based research is that it can have sustained impact because changes to the environment can be more enduring and can affect more than individual behaviour [23]. The growing interest in physical activity as a preventative health measure has given rise to ecological models which focus on the context of physical activity [21].

### COM-B framework

While the purpose of social ecological models is to understand the broad range of agency and factors contributing to health and wellbeing, COM-B provides a framework for behaviour change. Michie et al’s COM-B framework identifies the three key mechanisms of behaviour change as: motivation, capability and opportunity [20]. Motivation is viewed as an expression of an individual’s desire to perform an activity or to change behaviour whereas capability describes an individual’s capacity to perform an activity or to change behaviour which includes having the necessary physical ability, knowledge and skills. Opportunity captures external factors that enable or motivate behaviour which includes changes to the physical environment and social opportunities [20]. COM-B can add to the understanding of the mechanisms of behaviour change in social ecological models [20, 21, 27].

## Aims and methods

This paper draws on the literature on physical activity and sedentary behaviour to develop a social ecological model targeting office-based work and uses the COM-B framework to propose the mechanisms of behaviour change identified in the model. COM-B refers to capability, opportunity and motivation as the three conditions essential for behaviour change [20]. Social ecological models are complex nested models with individuals at the very centre. Individual factors include demographics, biological, psychological and family situation [21]. In this paper, we focus predominantly on context in developing the social ecological model as there is already a large body of work on the individual correlates of behaviour change [27, 29, 34, 35].

The first step in the development of a social ecological model for a specific context and a specific behaviour is the conceptualisation [36], involving a reflective process considering the factors involved, guided by the work of Sallis et al. and Giles-Corti et al. [21–23]. In this process, we scoped the extant literature on work and office-based physical activity and sedentary behaviour to conceptualise a social ecological model of office-based physical activity. We used a structured approach that focused on two different dimensions, the physical environment and the socio-cultural environment [23]. The nested nature of social ecological models involves consideration of multiple levels of agency from macro to micro. To build the model we scoped papers on physical activity/sedentary behaviour at work including activity associated with office work tasks, commuting and work break, and lunchtime activities. We included papers on the impact of the physical dimensions of office work settings, including the natural environment, the built environment, building design and ergonomics. For the social cultural dimension, we included papers on office policy and organisational and socio-cultural factors. To highlight the mechanisms of behaviour change inherent in the model, the first two named authors jointly mapped the model against the COM-B framework. Differences were resolved through discussion.

## Proposed social ecological model for office-based work

In social ecological models there are five domains of human activity; work, recreation, transport, household activities and sleep. The work domain is unique in that individuals have less personal agency. The physical activity of office workers is largely determined by the social, cultural and physical context of the workplace [37].

**Fig. 1:**
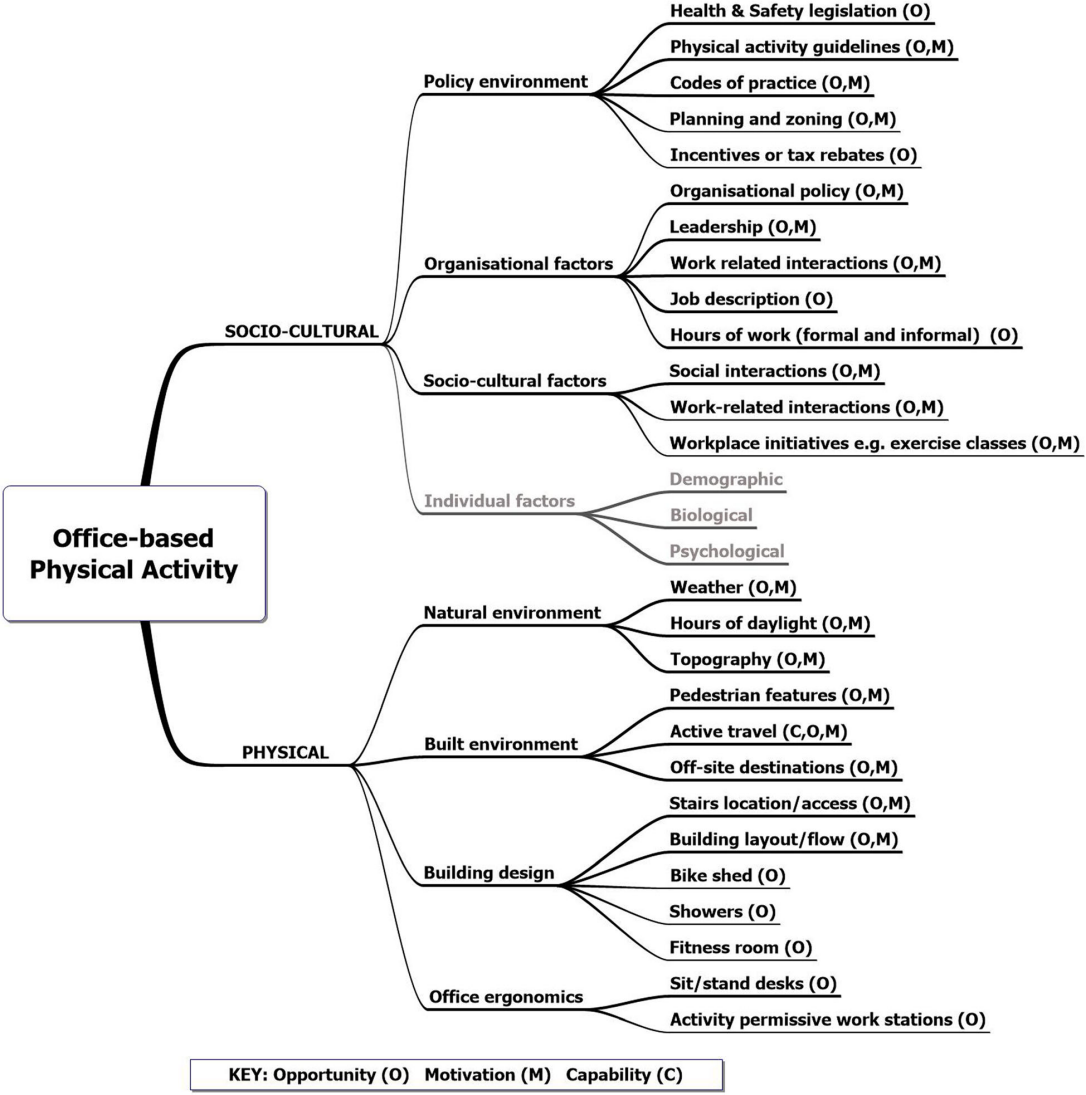
Social ecological model of office-based physical activity mapped to COM-B. Individual factors in the model come from Sallis et al. [21]

In the following sections we discuss the socio-cultural and physical dimensions of office-based work guided by frameworks developed by Sallis et al. and Giles-Corti et al. [21, 23] to propose a social ecological model for the specific context of office-based work using COM-B framework to highlight *opportunity, capability* and *motivation*. Both the dimensions of the ecological model and the COM-B mapping are summarised in Fig. 1.

### Socio-cultural dimensions.

Employment involves a contractual agreement stating employment conditions, outlining the rights, responsibilities and duties of employees. Employment and office-based work are complex socio-cultural constructs which include legal, mandatory and voluntary frameworks and workplace cultural practices such as unspoken expectations about how many hours we work a day. It also includes interactions with colleagues and social networks such as who we work with and with whom we share our breaks. In this section we look at three socio-cultural dimensions of office-based physical activity, ranging from macro to micro: policy, organisational, socio-cultural.

#### Policy environment

The political dimension of physical activity at work includes both mandatory frameworks for physical activity at work, such as workplace health and safety legislation, and voluntary frameworks such as policy, guidelines and best practice.

Mandatory frameworks that cover physical activity at work include federal or state legislation on health and safety at work. Legislation imposes responsibility and liability on organisations and workplaces [38]. Workplace health and safety law regulates the maximum number of hours worked, entitlement to rest breaks and rest days. Legislation impacts behaviour at work by regulating work hours to allow workers the *opportunity* to recover from the cumulative effect of work on both physical and mental health.

Voluntary frameworks include policy and guidelines. Policy at various levels from national and state level provide frameworks and guidance which impact organisations in different ways. For example, the use of incentives or tax rebates to encourage physical activity at work [38] create *capability* and *opportunity* for physical activity at work.

Increasing physical activity can also be achieved indirectly through changes in the built environment. Zoning changes can indirectly increase levels of physical activity [39]. Policy that impacts the built environment may result in improved attractiveness of outdoor spaces and more *opportunities* for physical activity around office buildings. These could include: changes in land use, zoning and access to public transport, support for active transport, improved street lighting and reduced traffic flow [38], changes which also impact *capability* and *motivation* to engage in physical activity in or around workplaces.

Guidelines on physical activity at work are published at many levels by statutory, non-statutory and advisory bodies. Such guidelines provide direction and information for employers and employees reflecting current best practice based on emerging research. Guidance provided to employers and employees impacts *capability* and *motivation* for physical activity through education and information about the benefits of physical activity at work.

#### Organisational factors

Employers control the nature of the work undertaken, the environment in which the work takes place, the number of hours worked, the time frame during which the hours are to be worked. All these, along with the nature of the occupation are key determinants of office-based physical activity at work. Seventy two percent of Australian jobs are in the service sector [40]. Changes in occupation impact the *opportunity* for physical activity at work. Growth in the proportion of service sector jobs over the last 50 years have resulted in population wide reductions in average daily occupation related energy expenditure and weight gain in the U.S. [41]. Research from the Netherlands also found that occupation determines the amount of sitting time. People working in computerisation, commercial services, transportation, banking insurance and government and judicial organizations sat significantly longer than the average worker [42].

Non-discretionary physical activity (read also inactivity) is dictated by job role, organisational practice and workplace culture [43, 44]. Full time office workers are expected to work ±7 h a day, predominantly sitting at a desk. Sedentary time at work adds to daily sedentary behaviour totals to increase the risk of disease associated with sedentary behaviour. For a fulltime worker, time spent at work comprises around 27% of waking hours a year, assuming 7 h a day at work. If you add in time spent commuting to work in cars or seated on public transport, then the proportion of time spent in sedentary behaviour increases again. Research comparing office, customer service, and call centre workers showed that call centre workers were both the most sedentary and spent more time in prolonged bouts of sedentary behaviour, than either office workers of customer services workers with customer service workers being the least sedentary of the three [14].

Organisations and workplaces not only have an obligation under law to provide for the safety and wellbeing of their employees, but they have a vested interest in reducing sick leave, productivity loss, workers compensation and disability management costs [45, 46]. At the corporate level, voluntary frameworks start with corporate health policy. Top down health policies provide the necessary framework, *motivation* and *opportunity* to unlock funding for physical activity initiatives and the *motivation* for workplaces to initiate and maintain corporate physical activity programs at work. Key strategies for successful corporate health policies include creating site specific health policies, thinking long term, having clear priorities, being consistent, communicating strategically, ensuring adequate resources, demonstrating leadership, engaging middle management, promoting employee participation, using incentives and awards and promoting active commuting [47]. Corporate policy is also an important enabler of *capability* for physical activity at work, through education and promotion, signage and awareness raising.

#### Socio-cultural factors

Socio-cultural support for office-based physical activity can build on the foundations of organisational health policy and health and safety at work, because to be effective, changes in safe work practices have to become an integral part of workplace culture [48]. The socio-cultural dimension of physical activity at work can support *capability* and *motivation* to engage in physical activity through health promotion, education, newsletters, signage, bulletin boards or initiatives such as corporate challenges and health promotion [49, 50]. Socio-cultural support for physical activity at work can also be achieved through leadership and example. Workers who perceived their co-workers and their managers as being physically active were more likely to be physically active at work [51]. Workplace culture can also negatively affect physical activity at work. It not uncommon for companies to have an unspoken culture of working late. Long working hours contribute to daily totals of sedentary behaviour and negatively impact workers health [52–54]. Another reason people move about an office is to talk to colleagues. A recent study in which office workers were relocated to a new building showed that expectations of quietness in the new building discouraged short non sedentary breaks in workflow occasioned by getting up to talk to colleagues or standing about having casual chats at colleagues’ desk [55].

### Physical dimensions

In general the physical dimensions, the setting of the physical activity, are less well researched [23]. This is less true for physical activity at work because of employer responsibilities under health and safety regulations and growing concerns over the negative health impacts of occupational sedentary behaviour. In this section we describe four different physical dimensions of office-based settings from micro to macro: ergonomics, building design and layout, the built environment and the natural environment.

#### Ergonomics

Research advises breaks from sitting every 30 min to reduce the risk of adverse health outcomes [56], however, the optimal duration of breaks in sedentary time or recommended activity (standing or sitting) during breaks is unknown. A commonly used cut point for breaks is a minimum duration of 1 min, a pragmatic option rather than one grounded in research [56, 57]. One minute breaks comprising a minimum of 50 movements per minute in the Diaz study and 100 movements/minute in the Healy study were beneficial in reducing all-cause mortality [56] waist circumference, BMI and triglycerides and 2-h plasma glucose [57]. Another study found that 2 min breaks that included light intensity exercise was better for cardio metabolic health than 2 min breaks that involved just standing which was no different to uninterrupted sitting [58]. Ergonomics has led to improvements in the design of office chairs and sit-stand desks to reduce the adverse impact of prolonged sedentary behaviour [59]. Sit-stand desks provide workers with the *opportunity* to reduce sedentary behaviour with standing breaks, however, to date the evidence of the effectiveness of activity permissive workstation (sit-stand desks, treadmill desks, etc.) is mixed. Chau et al. found that none of the 6 studies they reviewed showed significant differences in sedentary behaviour between intervention and control groups and MacEwen et al. found that while treadmill desks improved postprandial glucose, HDL cholesterol, and anthropometrics, sit-stand desks resulted in few changes [2, 60]. Similarly, a meta-analysis of activity permissive work stations showed a significant overall reduction in sedentary time of 77 min over an 8 h working day but non-significant results for health and work related outcomes [61].

#### Building design and layout

There has been little research on the impact of building design on physical activity and sedentary behaviour of office workers [55, 62]. Building design and floor layout can indirectly shape the organisation of work and the *opportunities* for incidental physical activity at work [63]. Incidental physical activity occurs in the completion of everyday tasks. In the office, incidental activity is typically short duration trips to and from communal facilities such as kitchens, bathrooms, printers, and meeting rooms [55, 64–66]. The amount of incidental activity a worker accrues in a work day depends largely on a worker’s desk location in relation to communal facilities.

*Opportunities* for physical activity at work can also be hardwired into the building design through the provision of facilities such as bike sheds, showers and a gym or space that can be used for exercise classes. Stairs are also an *opportunity* for including MVPA in daily work routines, however, importantly, the placement and visibility of the stairs relative to the ways in which people use space and move through the building impacts *motivation*, because convenience and efficiency are key considerations [55, 65]. The importance of stairwell placement is illustrated in a pre/post study of a workplace relocation. Office workers moved from one building to a purposed designed new building which resulted in a small but significant decrease in stair use attributable to change in stair placement and functionality and lack of connectivity between floors [55].

#### Built environment

The built environment surrounding the workplace is important for creating *opportunities* for sustained bouts of physical activity and potentially MVPA. Current guidelines recommend a minimum duration of movement of 10 min, which would be difficult to achieve inside a building [16–18]. The built environment determines *opportunity* and *motivation* for outdoor physical activity, influencing available commuting options, off-site destinations and pedestrian features. A recent systematic review showed that adult physical activity was significantly related to street/pedestrian connectivity and the proximity of non-recreational land use (cafes, shops, restaurants) [67]. Commuting, though not strictly work related, is nonetheless a non-discretionary activity associated with work unless you work from home. Long commutes to work can also add to daily sedentary behaviour however, commuting is also an *opportunity* for incidental physical activity, if only the walk from the car park or bus stop to the office. The built environment and local planning are big factors in determining the *opportunities* and *capability* for walking and cycling to work. Safety and ease of active transport options such as walking or cycling to work, proximity of public transport and car parks are all determinants of physical activity through commuting [68–70].

The presence of off-site destinations such as shops, food outlets, gardens or parks also provide both *opportunity* and *motivation* for physical activity during breaks at work. Research showed that the presence of offsite destinations within a radius of 800 m increases distance walked while at work [71] by affording *opportunities* for non-work related incidental physical activity such as going out for lunch or shopping [64]. *Motivation* to use outdoor space is also determined by pedestrian features such as footpaths and benches which can make open outdoor spaces more attractive and good lighting and traffic calming which can make walking safer [64].

#### Natural environment

Very few studies of physical activity account for climate or weather as separate variables, or even report, temperature or precipitation over the course of the data collection [33]. Changes in weather have most impact on discretionary behaviour and in particular *motivation* for MVPA. Understanding more about how climate and weather impacts the use of outdoor spaces at work is important [72, 73]. A systematic review of the effects of weather and seasonality on physical activity found that weather and seasonality are correlates of physical activity [33, 74]. To the best of our knowledge there is no research on the impact of weather on physical activity at work, but weather is likely to impact *motivation* for use of outdoor spaces while commuting and during work hours. There are a few studies showing the impact of weather on commuting. As might be expected, precipitation, temperature extremes and wind had significant impact on cycling to work [75, 76]. A study of student commuting also found that shortened daylight hours negatively affected cycle commuting [77].

Topography of the local environment can also influence *motivation*, *opportunities* and *capability* for MVPA. Hilly or steep precincts provide more incidental MVPA but may also impact *capability* and *motivation* to walk during breaks or active transport to work options such as walking and cycling. While there is little evidence of the impact of topography on physical activity at work, it has been found that hilly environments negatively impact on cycling and walking for household activities that could be reasonably walked or cycled and which did not involve carrying items (e.g. shopping) [78]. Rodríguez and Joo [79] found that, for students and staff of the University of North Carolina in Chapel Hill, slope negatively impacted propensity to walk or cycle. Similarly, other studies have found that hills have an overall negative impact of commuter cycling, but they are more appealing to experienced cyclists [80, 81]. While topography is not something that can change it nonetheless contributes to how people engage with active transport locally and it’s a factor that needs to be considered if research is being done across multiple sites.

#### Linking office-based physical activity opportunities to physical activity guidelines

Both the physical and socio-cultural dimensions combine to provide opportunities for physical activity at work. Table 1 summarises the opportunities against the current physical activity guidelines focussing highlighting the largely discretionary nature of physical activity in office-based settings.

**Table 1:** Mapping aspects of the office physical environment to current physical activity guidelines

Duration	Intensity	Location	Intention	Activity/ context	Description
Short	Light	Desk	Incidental	Standing	Sit-stand desk, talking to colleagues, printing, filing, standing whilst talking on the phone
Medium	Light	Desk	Discretionary	Standing	Sit-stand desk
Short	Light	Office Building	Incidental	Walking	Walking short distances (stop/start): Trips to and from bathroom, kitchen meeting rooms
Short/ Medium	Light	Precinct, Neighbourhood	Incidental	Commuting	Walk to car park or transit point
Medium/ Long	Light MVPA	Precinct, Neighbourhood	Discretionary	Commuting	Walking to work^a^
Medium/ Long	MVPA	Precinct, Neighbourhood	Discretionary	Commuting	Cycling to work^a^
Short	MVPA	Building	Incidental	Climbing stairs	Using the stair as an efficient way for getting from A to B
Short/ Medium	MVPA	Building	Discretionary	Climbing stairs	Using the stair for increasing PA
Medium	MVPA	Building	Discretionary	Gym	Gym, exercise classes, jogging
Medium	Light MVPA	Precinct, neighbourhood	Discretionary	Brisk walking	Walking breaks, walking groups
Medium	Light	Precinct, Neighbourhood	Discretionary	Work breaks	Walks out doors during breaks, walking to local shops and cafes, restaurants

^a^Local area (reasonable cycling distance 32 min – walking 22 min for FT worker [82]

## Conclusions

High levels of sedentary behaviour associated with all-cause mortality and chronic disease morbidity are now endemic in developed countries. Sedentary behaviour at work is a contributing factor given that on working days, half our waking hours are spent at work. Growth in service sector and computer-based employment combined with the rising age of retirement in developed countries are set to increase occupational sedentary behaviour and the associated risks. In this article, in line with guidelines on physical activity which recommend both daily/weekly targets of MVPA as well as reduction in sedentary behaviour and especially prolonged sedentary behaviour, we reviewed socio-cultural and physical factors impacting physical activity for office-based workers to develop a social ecological model to inform interventions aimed at reducing sedentary behaviour and increasing physical activity for office workers. We then mapped each option against the COM-B framework to identify the underpinning mechanisms of behaviour change (opportunity, capability and motivation).

Behaviour change is a focal element of many interventions to increase physical activity, however, research into public health interventions has shown that a broader approach based on social ecological models is more successful than targeting individual behaviour alone [25, 83]. This is particularly true for physical activity at work because of the non-discretionary nature of sedentary behaviour of office workers. The organisation and the workplace largely determine the capability, opportunity and motivation for physical activity at work. A systematic review comparing the effectiveness of different types of interventions found that interventions that included organisational (e.g. steering committees, senior management endorsement, action days and gymnastics breaks) and environmental elements (e.g. signage, maps, staircase promotion, walking tracks) reported better results than interventions which focussed on behavioural change and information only [84].

This paper contributes to the literature by proposing a context specific social ecological model for office-based work aimed at reducing sedentary behaviour and enhancing physical activity [23] and identifying the mechanisms for behaviour change inherent in the resulting social ecological model. The model can help inform the development of more complex and context specific work place interventions to reduce sedentary behaviour and increase physical activity for office-based workers.

Being more inclusive of a range of different factors involved in measuring change or monitoring physical activity in office-based setting is a challenge for the collection of data, however, the field of physical activity research is changing. The last decade has seen the use of research grade accelerometers being used to measure physical activity and more particularly sedentary behaviour of office workers. Data collection is typically around 7 days [6, 7, 62]. A recent study by Mullane et al. [85] investigated the social ecological correlates of sedentary using ActivPAL inclinometers (7 days) and two online surveys. Results showed that physical activity was positively related to lunchtime walking and that talking to colleagues was negatively correlated with prolonged sitting. Furthermore, open plan or shared offices were less associated with prolonged sitting than private offices.

Advances in technology and connectivity present new opportunities for understanding physical activity in context-based on growth in consumer wearables, use of mobile phones and the internet of things (IOT), devices which can be used to collect data from individuals and the environment.

First, the improved reliability and accuracy of consumer activity trackers means that these relatively low cost devices are increasingly being used for longitudinal research to understand physical activity in free living environments with large sample sizes over longer periods of time [86–88]. The collection of big data on physical activity opens opportunities for research that can investigate how for example, job descriptions, seniority or socio-demographic factors impact incidental and discretionary physical activity at work. Longitudinal temporal data also allows researchers to develop profiles of how physical activity is distributed over time to understand patterns of behaviour [14, 33, 88, 89]. Understanding how physical activity is distributed over time is very relevant to research on physical activity at work. Timing of breaks in sedentary behaviour is important for achieving health benefits of sit-stand desks and other behaviour change designed to reduce the length of bouts of sedentary behaviour. Work activities are time sensitive, so it is important to understand because opportunities for physical activity are largely regulated by workplace organisation and culture such as hours of work and timing of meetings and breaks.

Second, the ubiquity of mobile phones and the growth in the IOT will facilitate the collection of contextual data to supplement data from consumer activity trackers [88]. While the use of contextual data to add to the understanding of physical activity is still new [33, 88], increasingly global positioning systems (GPS) and other environmental sensors are being used to track movement [90]. One of the issues with research on changes in infrastructure or the built environment is the difficulty of measuring and evaluating change [91]. The addition of environmental and location data with data from smart phones or commercial activity trackers can create new insights into how context influences physical activity [88, 92, 93] and can help inform workplace design and changes to the built environment to better facilitate both incidental and discretionary physical activity. It is also possible to use data from activity trackers and the IOT to profile or rate buildings based on the opportunities for physical activity for specific buildings and the local built environment, to understand more about designing activity permissive office environments [eg. 62].

The collection of digital data on physical activity and context using consumer activity trackers, mobile phones and sensors in the environment can lead to the creation of large datasets or big data. Big data with high levels of granularity can address different research questions [87, 94–96], however it can also change the nature of research which is likely to become more multi-disciplinary given the new challenges of data storage, management and analysis.

Social ecological models and COM-B together, draw attention to the dynamic interplay of factors both socio-cultural and physical that can contribute to facilitating behaviour change to increase physical activity at work. The social ecological model is a useful framework for thinking holistically about physical activity in office-based settings to guide the design and collection of contextual data and inform different research questions [21, 27, 97]. To increase levels of physical activity in office-based settings, it is important for interventions not to rely as strongly on individual motivation for behaviour change but to incorporate changes to the broader social ecological and physical context to build capability and create opportunities for more sustainable change.

## Data Availability

Not applicable.

## Abbreviations

COM-B: Capability motivation opportunity - behaviour
GPS: Global positioning system
IOT: Internet of things
METs: Metabolic equivalents
MVPA: Moderate to vigorous physical activity
